# Adherence and effectiveness of an emotion-based psychodynamic online self-help during and after inpatient and day-care psychotherapy: Results of a naturalistic study

**DOI:** 10.3389/fpsyt.2023.1027118

**Published:** 2023-01-20

**Authors:** Jan Becker, Adina Kreis, Theresa Schorch, Anna Mayer, Angeliki Tsiouris, Manfred E. Beutel, Rüdiger Zwerenz

**Affiliations:** ^1^Institute of Teachers’ Health, University Medical Center of the Johannes Gutenberg-University Mainz, Mainz, Germany; ^2^Department of Psychosomatic Medicine and Psychotherapy, University Medical Center of the Johannes Gutenberg-University Mainz, Mainz, Germany

**Keywords:** internet intervention, online self-help, adherence, psychosomatic therapy, psychotherapy, inpatient treatment, routine care, emotion-focused therapy

## Abstract

**Background:**

Internet-based mental health interventions are considered effective in providing low-threshold support for people with mental health disorders. However, there is a lack of research investigating the transferability of such online programs into routine care settings. Low treatment adherence and problems with technical implementation often limit a successful transfer into clinical routines. This naturalistic study aims to identify influencing factors on program adherence in patients who participated in an online intervention during inpatient or day-clinic psychotherapeutic treatment.

**Methods and findings:**

In a naturalistic study, we investigated the transferability of the transdiagnostic psychodynamic online self-help program *KEN-Online*, which includes eight consecutive units. Between May 2017 and October 2018, patients who received inpatient or day-clinic psychotherapeutic treatment at the Department of Psychosomatic Medicine and Psychotherapy in the University Medical Center Mainz have been offered to use *KEN-Online*. Of the *n* = 749 patients who were admitted to the clinic, *n* = 239 patients (32%) registered for participation in *KEN-Online*. While 46.9% of the participants did not complete any unit (inactive participants), 53.1% completed at least the first unit (active participants). Age, number of diagnoses, and symptom severity were associated with (in)active participation. Adherence decreased over time resulting in only 17 participants (7.6%) who completed all units. None of the sociodemographic and medical characteristics proved to be significant predictors of adherence. Analyses of effectiveness showed a significant reduction of anxiety and depression in active participants in the course of participation, with higher improvements in participants that completed more than half of the units.

**Conclusion:**

Adherence to the online self-help program *KEN-Online* was lower in the naturalistic setting than in a previous clinical trial, but was still associated with greater program effectiveness. Adherence-promoting measures are crucial to increase the effectiveness of such interventions in clinical settings.

## 1. Introduction

With increasing prevalence of mental health disorders worldwide, health care is facing an unmet need for treatment. Over the course of 1 year, 27.8% of the adult population in Germany suffer from mental illness (1). The number of work disability days due to mental health issues is constantly increasing (2). However, many of those affected do not get sufficient treatment. Internet-based mental health interventions are a promising approach to reduce the existing treatment gap. A variety of web-based interventions in different treatment contexts have shown medium to high efficacy for a range of mental health disorders (3, 4). Both cognitive behavioral and psychodynamic interventions proved effective (5–7).

However, concerns are raised due to the difficulty in transferring interventions from experimental conditions into routine clinical settings. While structural and procedural aspects, as well as the stakeholders’ and practitioners’ attitudes toward internet interventions all appear to play a role in implementation of internet interventions into primary care, low treatment adherence has shown to be one of the main limiting factors on the part of the user (8, 9). Treatment adherence, which describes the degree to which an individual participates in online interventions, was found to vary considerably between different studies (10). In their systematic review, Christensen et al. (10) reported adherence rates from 50 to 70%. While low adherence is a common problem in research trials investigating internet interventions, attrition rates are even higher when programs are implemented into routine care (11–13).

However, as with any kind of treatment, for internet-based interventions to be effective, patients have to engage in the programs. While patients who do not fully complete the intervention still benefit (12, 14), treatment effects are considerably stronger for those who complete a higher number of modules or fully complete an intervention (14, 15). Considering the crucial role of adherence for the effectiveness of internet interventions, it is important to understand patient characteristics that may influence treatment adherence. However, respective studies yield inconsistent findings. While age is one of the most examined predictors, findings concerning its role regarding intervention adherence are mixed. Some studies found older age associated with higher treatment adherence (16). Others, however, had contrary results (10, 17). Similarly, the exact extent to which the participants’ gender or level of education influences intervention usage remains unclear due to inconsistent findings. Several studies showed female gender to be associated with higher adherence, and male gender to be related with low adherence (16, 17), whereas Christensen et al. (10) could not find a relationship between gender and adherence. With regard to the level of education, a meta-analysis by Karyotaki et al. (16) found that low education levels increased the risk of dropping out from treatment, while the majority of studies assessed in the systematic review by Beatty and Binnion (17) did not find a significant relationship with adherence. Besides sociodemographic variables, various other factors such as inadequate technical skills or the lack of sufficient time might play an additional role in progressing with and completing a program (17–19).

In conclusion, findings regarding the association between participant characteristics and adherence are inconsistent. Moreover, the limited transferability of study results to routine care settings calls for further investigation of the association of program adherence and effectiveness in routine care patients. Only then can internet-based mental health interventions be successfully incorporated into psychiatric healthcare.

*KEN-Online* (“Die Kraft der eigenen Emotionen Nutzen” in German and “Use the power of your own emotion” in English) is a transdiagnostic psychodynamic internet-based self-help program that aims to strengthen emotional awareness (20). It has been examined regarding feasibility and efficacy in a previous randomized controlled study and proved effective in reducing symptoms of depression (*d* = 0.60), as well as increasing emotional competence (*d* = 0.49) and quality of life (*d* = 0.53) (14). While acceptance and overall satisfaction were good, a steady decline in participation could be observed over the course of the program. Only 36% participated throughout the trial completing at least four of the total eight units.

In 2017, *KEN-Online* was integrated into inpatient and day-care psychotherapy at the Department for Psychosomatic Medicine and Psychotherapy at the University Medical Center Mainz. The goal of this study was to identify influencing factors on program adherence in routine care patients who participated in *KEN-Online* during and/or after inpatient and day-clinic psychotherapeutic treatment. For this purpose, we set out to (1) compare sociodemographic variables and disease-related characteristics of patients with varying degrees of treatment adherence, and (2) examine the influence of treatment adherence on the effectiveness of the treatment concerning symptoms of anxiety, depression, and emotional competence.

## 2. Materials and methods

### 2.1. Ethics statement

An ethics vote was not required for the study. Since the intervention is part of the regular treatment program, according to the Ethics Committee of the Federal State of Rhineland Palatinate (Germany), the Rhineland-Palatinate Hospital Act can be applied, which in turn allows retrospective evaluation of anonymized patient data without additional written consent.

### 2.2. Recruitment

A naturalistic study design without a control group was chosen for the study to obtain results transferable to everyday clinical practice. Accordingly, all patients who attended the Department of Psychosomatic Medicine and Psychotherapy in the University Medical Center Mainz between May 2017 and October 2018 have been offered to use *KEN-Online* during and after their inpatient or day-clinic treatment. The offer was made shortly after admission to the clinic as well as during ongoing therapy. Treating therapists briefly informed their patients of the possibility to use KEN-Online in a therapy session. Participation in *KEN-Online* was voluntary. Interested patients could request the login data *via* e-mail. The prerequisite for inclusion in the analysis was a minimum length of stay in the clinic of two weeks and the presence of at least one mental ICD-10 diagnosis.

### 2.3. Intervention

The text-based transdiagnostic intervention is based on the principles of mindfulness (21) and affect phobia (22) and is described extensively in Becker et al. (20). Over the course of eight consecutive units, participants go through four steps: (1) enhancing awareness of their emotions and related defenses, (2) regulating emerging anxiety when approaching feared emotions, (3) fully experiencing their emotions and further (4) mindfully expressing their emotions to other people. Each unit includes multiple-choice and writing tasks, some are accompanied by text and audio-exercises. Standardized questionnaires (see section “2.4. Questionnaires and data collection”) are integrated into the program to regularly record and monitor relevant psychological symptoms.

The program is routinely offered to inpatients and day-care patients of the Department for Psychosomatic Medicine and Psychotherapy at the University Medical Center Mainz as an add-on to regular treatment. Inpatient treatment and day-care are offered as multimodal treatment consisting of two to three individual therapy sessions per week, two weekly sessions of art therapy, up to two sessions of body-oriented therapy, and up to three sessions of group therapy. The duration of treatment is typically 8–12 weeks. While the focus of the clinic is psychodynamic, treatment integrates different schools and modalities, including educational elements regarding pathogenesis and maintenance of the disorder, and specific modules (e.g., relaxation training or physiotherapy) tied to the individual needs of the patients. If interested, patients receive access to KEN-Online during treatment and can integrate relevant topics of the self-help intervention into their therapy sessions. While study participants in the feasibility study received weekly feedback on completed tasks (14, 20), contact with online therapists in routine psychotherapeutic setting is limited to technical support. Patients can access the intervention with their personal laptops or mobile devices free of charge. Patients had access to the intervention for 12 months, with the option to prolong if they felt the need to.

### 2.4. Questionnaires and data collection

Emotional competence was measured with the 27-item Emotion-Regulation Skills Questionnaire (ERSQ) (23). Item (e.g., “*In the last week I knew what my feelings mean*.”) answers range from 0 = “*not at all*” to 4 = “*(almost) always*” and are summed up to an index ranging from 0 to 108. Internal consistency is very high (Cronbach alpha = 0.95). Depression was measured with the Patient Health Questionnaire (PHQ-9) (24). The scores (ranging from 0 = “not at all” to 3 = “*nearly every day*”) of the nine items (e.g., “*Over the last 2 weeks, how often have you been bothered by little interest and pleasure in doing things?*”) are added to a sum score ranging from 0 to 27. Sum scores can be categorized as minimal (below 5), mild (5–9), moderate (10–14), and severe (above 14) depressive symptoms. Internal consistency is good (Cronbach alpha = 0.81). Anxiety was assessed with the General Anxiety Disorder (GAD-7) (25) questionnaire (e.g., “*Over the last 2 weeks, how often have you been bothered by feeling nervous, anxious or on edge?*”), utilizing the same Likert scale as the PHQ-9 with sum scores ranging from 0 to 21. Severity cut-offs for minimal, mild, moderate, and severe anxiety symptoms are analogous to the PHQ-9. Internal consistency is good (Cronbach alpha = 0.81). Somatic symptoms were measured with the Somatic Symptom Scale (SSS-8) (26), a reliable (Cronbach alpha = 0.77) self-report measure covering gastrointestinal, pain, fatigue, cardiopulmonary, and general somatic symptoms burden over the past 7 days (e.g., “*During the past 7 days, how much have you been bothered by stomach or bowel problems?*”). Each item can be answered on a Likert scale from 0 = “*not at all*” to 4 = “*very much.*” The sum score of the eight item index ranges between 0 and 32 points. Symptoms of depersonalization and derealization were screened with the 2-item (e.g., “*Out of the blue, I feel strange, as if I were not real or as if I were cut off from the world*.”) version of the Cambridge Depersonalization Scale (CDS-2) (27). Both items (ranging from 0 = “*not at all*” to 3 = “*nearly every day*”) are added for a sum score between 0 and 6. Internal consistency is very good (Cronbach alpha = 0.92).

Personality traits were measured with the brief form of the personality inventory for DSM-5 (PID-5-BF) (28). It consists of 25 items (e.g., “*I get emotional easily, often for very little reason*.”) rated on a 4-point Likert scale ranging from 0 = “*Very False or Often False*” to 3 = “*Very True or Often True.*” Five items each measure one of five higher-order facets (Negative Affect, Detachment, Antagonism, Disinhibition, Psychoticism). Reliability is acceptable (Cronbach alpha = 0.62–0.66). Psychosocial stressors were assessed with the stress module of the Patient Health Questionnaire (PHQ-Stress) (29). It consists of ten items, asking how much one has been affected by different complaints (e.g., worries about health, stress at work or school) during the last 4 weeks (range 0 = “*not at all bothered*” to 2 = “*bothered a lot*”), which are added up to a sum score between 0 and 20. Internal consistency is acceptable (Cronbach alpha = 0.64). Depression and anxiety were measured additionally with the Patient Health Questionnaire-4 (PHQ-4) (30), a shortened combination of the PHQ-9 and GAD-7. The PHQ-4 consists of four items, two measuring depressive and two anxiety symptoms. Internal consistency is high (Cronbach alpha = 0.86).

Adherence was operationalized by the number of units completed by every participant. The number of completed units and logins was determined based on objective data collected within the system that provided the intervention.

Data was collected at admission (T0) and discharge (T1) as well as at the beginning of the intervention (T2), after completing the last unit (T3), and every week during the intervention (resp. at the next login after 7 days without a login; T-weekly). At T0 and T1, data from the clinical documentation were used. For our analyses, we used age, sex, partnership, family status, employment, and the number of ICD-10 diagnoses as sociodemographic variables. In addition, the following outcome measures were part of the documentation and used in our study: personality traits (PID-5-BF), anxiety (GAD-7), depression (PHQ-9), depersonalization and derealization (CDS-2), somatic complaints (SSS-8), and psychosocial stress factors (PHQ-Stress). At T2 emotional competence (ERSQ) was measured. At T3, emotional competence (ERSQ), depression (PHQ-9), anxiety (GAD-7), and somatic complaints (SSS-8) were assessed. Furthermore, anxiety and depression were measured with the PHQ-4 at T2, T3, and T-weekly.

### 2.5. Data analysis

All analyses were conducted with IBM SPSS Statistics version 26. For the sample description and overall adherence, descriptive data and frequencies were analyzed.

We defined groups based on their levels of participation and adherence: first, we differentiated between inactive participants (no unit completed) and active participants (one to eight units completed). Further, we divided the group of active participants in non-completers (one to seven units completed) and completers (all units completed). To examine differences between different types of adherence, inactive participants were compared with active participants, and non-completers were compared with completers using group comparisons (*t*-tests and chi-square tests) with sociodemographic variables and outcome measures from the clinic documentation (T0/T1) as dependent variables. To examine the influence of adherence on depressive symptoms and anxiety, an analysis of covariance (ANCOVA) was performed in the group of active participants with the last T-weekly measurement of the PHQ-4 as dependent variable, adherence as independent variable and the sum score of the PHQ-4 at T2 as covariate.

Overall effectiveness of *KEN-Online* was assessed with a *t*-test for dependent samples (active participants; T2 and last T-weekly measurement) with the PHQ-4 as dependent variable. Additionally, effectiveness of *KEN-Online* for completers was calculated using *t*-tests for dependent samples. The outcomes collected at T2/T3 each served as the dependent variable. Effect sizes were calculated where applicable. Missing values were not replaced.

## 3. Results

### 3.1. Sample description

Between May 2017 and October 2018, 749 patients were admitted to an inpatient or day-clinic treatment in the Department for Psychosomatic Medicine and Psychotherapy of the University Medical Center Mainz. 330 patients were treated as day-care inpatients and 337 as inpatients, 82 patients were treated in both settings. Of the 749 patients admitted, 239 patients (32%) registered for participation in *KEN-Online*. As sociodemographic and questionnaire data on mental health from the basic documentation were not available, *n* = 15 participants were excluded from the analyses, resulting in a total of *n* = 224 participants. Sociodemographic and medical data of the sample can be obtained from Table 1. Mean age in the sample was 39.13 years (*SD* = 13.34), with a range from 16 to 74 years. Slightly more women (57.1%) took part in the intervention. Up to five mental diagnoses were coded for every participant, with two and three diagnoses being most prevalent (29.0% and 29.9%). Four diagnoses were reported by 17.0%, and less than 15% had one or five diagnoses. The most common mental disorders were affective disorders (84.8%) followed by anxiety (50.0%) and personality disorders (40.6%).

**TABLE 1:** Sociodemographic and medical data of the sample.

Variables		*N* ^c^	*n*	*%*	*M*	*SD*
**Sex**						
	Male		96	42.9		
	Female		128	57.1		
**Age**		224			39.13	13.34
**Partnership**						
	Yes		121	54.0		
	No		96	42.9		
**Education**						
	Still at school		2	0.9		
	No, lower or other graduation		21	9.4		
	Middle secondary		59	26.3		
	Higher secondary		135	60.3		
	Other		2	0.9		
**Marital status**						
	Single		132	58.9		
	Married		69	30.8		
	Separated, divorced, widowed		17	7.6		
**Employment**						
	Employed		127	56.7		
	Unemployed		36	16.1		
	Other		21	9.4		
**Psychiatric diagnoses**		224			2.81	1.18
	Affective disorders (F30-F34)		190	84.8		
	Anxiety disorders (F40–F41)		112	50.0		
	Personality disorders (F60–F69)		91	40.6		
	Depersonalization-derealization disorder (F48.1)		51	22.8		
	Somatoform disorder (F45)		60	26.8		
	Other disorders (including F50, F1x)		66	29.5		
**Symptom severity**	Maladaptive personality traits (PID-5-BF)^a^		105		20.79	8.91
	Anxiety (GAD-7)^a^		204		11.65	4.69
	Depression (PHQ-9)^a^		195		14.67	5.28
	Psychosocial stressors (PHQ-Stress)^a^		182		8.25	3.86
	Somatic symptoms (SSS-8)^a^		205		12.20	5.94
	Depersonalization-derealization (CDS-2)^a^		208		2.12	2.33
	Emotional competence (ERSQ)^b^		161		49.24	18.06

^a^At admission to the clinic.

^b^At the start of the intervention.

^c^Missing percentages to 100 show missing data, with the exception of multiple answer questions.

### 3.2. Adherence

**FIGURE 1:**
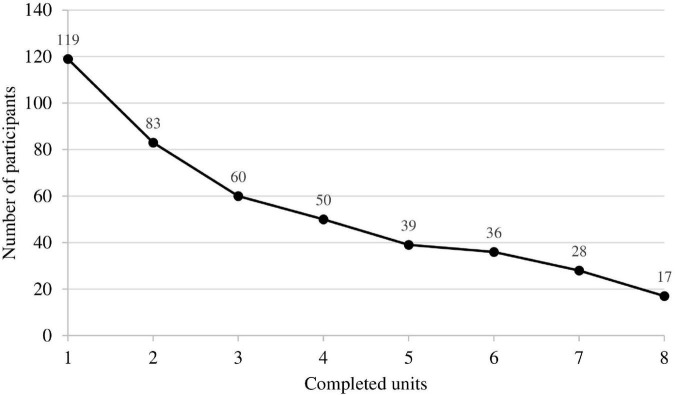
Number of participants who completed the respective number of units.

Among the 224 registered participants, 105 participants did not complete any unit (inactive participants; 46.9%), while 119 completed at least the first unit (active participants; 53.1%). Of the 224 registered participants 102 completed one to seven units (non-completers; 45.5%), 50 (22.3%) completed half of the intervention (four units). Finally, 17 participants completed all units (completers; 7.6%). The number of completed units can be seen in Figure 1.

To examine if inactive participants showed differences in certain characteristics, they were compared with active participants (Table 2).

**TABLE 2:** Descriptive and test-statistics of the comparison between inactive and active participants.

Variables	Inactive participants			Active participants			Test statistics		
	***N***			***N***			**χ^2^ *(df)***	***p***	**φ**
Sex	105			119			1.17 (1)	0.28	0.72
Male	49			47					
Female	56			72					
Partnership	104			113			3.68 (1)	0.055	0.13
Yes	65			56					
No	39			57					
Marital status	104			114			0.43 (2)	0.81	0.05
Single	64			68					
Married	31			38					
Separated, divorced, widowed	9			8					
Employment	93			91			0.15 (2)	0.93	0.03
Employed	63			64					
Unemployed	19			17					
Other	11			10					
	***N***	***M***	***SD***	***N***	***M***	***SD***	***t (df)***	***p***	***d***
Age	105	37.2	12.68	119	40.84	13.73	–2.06 (222)	0.04	0.28
Number of psychiatric diagnoses	105	3.02	1.18	119	2.62	1.16	2.54 (222)	0.01	0.34
**Symptom severity**									
Maladaptive personality traits (PID-5-BF)^a^	53	20.49	9.43	52	21.1	8.43	0.35 (103)	0.73	0.07
Anxiety (GAD-7)^a^	94	12.3	4.79	110	11.09	4.55	1.85 (202)	0.07	0.26
Depression (PHQ-9)^a^	91	14.46	5.65	104	14.86	4.96	0.53 (193)	0.60	0.08
Psychosocial stressors (PHQ-Stress)^a^	84	8.71	4.02	98	7.85	3.70	1.52 (180)	0.13	0.23
Somatic symptoms (SSS-8)^a^	95	12.53	6.11	110	11.91	5.80	0.75 (203)	0.46	0.11
Depersonalization-derealization (CDS-2)^a^	98	1.98	2.32	110	2.25	2.34	0.84 (206)	0.40	0.12
Emotional competence (ERSQ)^b^	58	50.57	18.77	103	48.5	17.69	0.70 (159)	0.49	0.12

^a^At admission to the clinic.

^b^At the start of the intervention.

Analyses of the sociodemographic variables age, sex, partnership, marital status, and employment as dependent variables revealed significant differences between inactive participants and active participants only concerning age. Active participants were significantly older, *t*(222) = –2.06, *p* = 0.04, *d* = 0.28. In addition, the analysis revealed an almost significant difference in regard to partnership with active participants being less likely in a stable partnership, χ^2^(1) = 3.68, *p* = 0.055, φ = 0.13. Furthermore, we examined possible differences between inactive participants and active participants concerning the number of diagnoses and symptom severity at the time of their admission to the clinic. The analysis revealed that inactive participants had significantly more mental diagnoses, *t*(222) = 2.54, *p* = 0.012, *d* = 0.34. Besides a statistical significant higher anxiety score of inactive participants, no other significant differences in symptom severity were observed between inactive participants and active participants.

To further understand differences in adherence, non-completers (*n* = 102) were compared with completers (*n* = 17). None of the sociodemographic variables revealed a significant group difference (Table 3). The same applies to number of psychiatric diagnoses and symptom severity. An almost significant difference existed concerning emotional competence, *t*(101) = 1.87, *p* = 0.07, *d* = 0.51. Completers reported higher emotional competence at the beginning of the intervention.

**TABLE 3:** Descriptive and test-statistics of the comparison between non-completers and completers.

Variables	Non-completers			Completers			Test statistics		
	***N***			***N***			**χ^2^ (*df*)**	***p***	**φ**
Sex	102			17			0.84 (1)	0.36	0.84
Male	42			5					
Female	60			12					
Partnership	96			17			0.05 (1)	0.82	0.21
Yes	48			8					
No	48			9					
Marital status	97			17			0.56 (2)	0.76	0.07
Single	59			9					
Married	31			7					
Separated, divorced, widowed	7			1					
Employment	77			14			3.81 (2)	0.15	0.21
Employed	52			12					
Unemployed	17			0					
Other	8			2					
	***N***	***M***	***SD***	***N***	***M***	***SD***	***t (df)***	***p***	***d***
Age	102	40.59	13.51	17	42.35	15.36	0.49 (117)	0.63	0.13
Psychiatric diagnoses	102	2.63	1.17	17	2.59	1.12	0.13 (117)	0.90	0.03
**Symptom severity**									
Maladaptive personality traits (PID-5-BF)^a^	45	20.89	8.59	7	22.43	7.76	0.45 (50)	0.66	0.18
Anxiety (GAD-7)^a^	93	10.9	4.56	17	12.12	4.50	1.01 (108)	0.31	0.27
Depression (PHQ-9)^a^	88	14.95	4.93	16	14.31	5.23	0.48 (102)	0.64	0.13
Psychosocial stressors (PHQ-Stress)^a^	83	7.92	3.84	15	7.47	2.88	0.43 (96)	0.67	0.12
Somatic symptoms (SSS-8)^a^	93	11.85	6.08	17	12.24	4.00	0.25 (108)	0.80	0.07
Depersonalization-derealization (CDS-2)^a^	94	2.22	2.38	16	2.44	2.13	0.35 (108)	0.73	0.10
Emotional competence (ERSQ)^b^	87	47.11	17.40	16	56.00	17.89	1.87 (101)	0.07	0.51

^a^At admission to the clinic.

^b^At the start of the intervention.

### 3.3. Effectiveness

Analysis of overall effectiveness in active participants (Table 4) revealed a significant reduction of anxiety and depression (PHQ-4) between T2 and the last T-weekly measurement [*t*(102) = 5.19, *p* < 0.001, *d* = 0.51].

**TABLE 4:** Descriptive and test-statistics of the pre-post inner subject comparison of the primary and secondary outcomes.

Variables	T1			T2			Test statistics		
	***N***	***M***	***SD***	***N***	***M***	***SD***	***t (df)***	***p***	***d***
**Regular participants**									
PHQ-4^a^	103	7.00	3.02	103	5.46	3.32	5.19 (102)	<0.001	0.51
**Completers**									
PHQ-4^a^	17	7.53	2.55	17	4.29	3.06	4.46 (16)	<0.001	1.08
ERSQ	7	52.29	14.49	7	78.29	14.26	2.76 (6)	0.03	1.04
PHQ-9	7	12.71	5.96	7	9.57	4.28	1.90 (6)	0.11	0.72
GAD-7	7	10.71	3.99	7	9.00	3.16	1.14 (6)	0.30	0.43
SSS-8	7	10.86	3.63	7	9.57	4.47	0.89 (6)	0.41	0.34

^a^In this analysis we used the last t-weekly measurement instead of T2, which did not necessarily take place at the end of the intervention.

Among completers, anxiety and depression symptoms (measured with the PHQ-4) were also reduced significantly [*t*(16) = 4.46, *p* < 0.001, *d* = 1.08]. Additionally, completers showed higher emotional competence at T3 compared to T2, *t*(6) = 2.76, *p* = 0.03, *d* = 1.04. The analysis showed no significant reduction of depression symptoms {measured with the PHQ-9 [*t*(6) = 1.90, *p* = 0.11, *d* = 0.72]} between T2 and T3. Furthermore, completers showed no significant reduction in anxiety symptoms {measured with the GAD-7, [*t*(6) = 1.14, *p* = 0.30, *d* = 0.43]} and somatic symptoms {measured with the SSS-8, [*t*(6) = 0.89, *p* = 0.41, *d* = 0.34]} between T2 and T3 (Table 4).

The ANCOVA revealed a significant influence of adherence on anxiety and depression in active participants [*F*_(8,__94)_ = 3.07, *p* = 0.006, *d* = 0.96].

**FIGURE 2:**
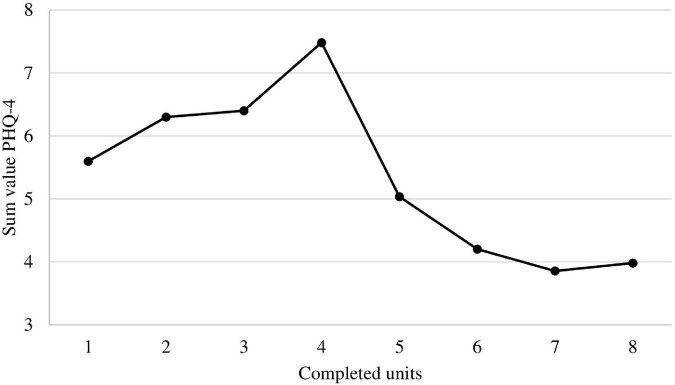
Mean of the PHQ-4 in relation to program adherence (number of completed units). Note that the y-axis was cut to obtain more details on the graph.

Figure 2 shows the mean (score) of the PHQ-4 in relation to program adherence (number of completed units). The graph shows that participants with less than five completed units reported notably higher values in their last PHQ-4 questionnaire compared to participants with five to eight completed units.

## 4. Discussion

The aim of the present naturalistic study was to examine the emotion-based psychodynamic online intervention *KEN-Online* regarding its utilization and adherence among patients as well as its effectiveness in a routine psychotherapeutic setting. Since the efficacy of *KEN-Online* was relatively low in a prior randomized controlled trial (RCT) despite high satisfaction values, this naturalistic observational study examined whether and how *KEN-Online* is accepted and utilized during inpatient and day-clinic psychosomatic psychotherapy.

Although the analysis showed that adherence was lower than expected, still more than half of the registered users (53%) completed at least one unit and about 30% finished more than half of the units. However, the proportion of completers was very low at just under 10%. In comparison, 86% of the participants in the previous study completed the first unit, 36% completed half of the intervention and 22% completed all eight units of the intervention (14). Accordingly, adherence was lower after implementation of the intervention in standard care compared to the RCT in our feasibility study (14, 20). In contrast to the feasibility study (20), no feedback from an online therapist was offered in the present study. The connection between therapeutic feedback and adherence is still partly inconsistent, but most studies so far have confirmed that online self-help programs without any kind of guidance show less adherence than guided interventions (31). In contrast, a recent meta-analysis of digital interventions for anxiety disorders did not report any superiority in the effectiveness of guided interventions compared to unguided programs (32). While the participants in the feasibility study were informed about the study, the intervention and the study website at an information event (20), the patients who used *KEN-Online* in this study only received information from their therapist and were not instructed in detail about the utilization of *KEN-Online*, which could also be an explanation for the low acceptance of the intervention. In general, however, it can be said that many internet-based interventions face the problem of low adherence, whereby unaccompanied interventions without therapeutic feedback are particularly affected (16, 17). In addition, a study promoting physical activity showed, that adherence to self-help programs is significantly greater if the intervention is video-based (33) than it is to mainly text-based interventions like *KEN-Online*. In another study, Walthouwer et al. (34) were able to show that the video-based version of their intervention to prevent obesity was rated and accepted significantly better by participants when directly compared to the text-based version. It is possible that, especially at a time when visual forms of communication are becoming more and more important in everyday life (35), the effort required to read the text-based units of the *KEN-Online* intervention is too high for patients.

To analyze patient characteristics influencing adherence, inactive participants were compared to active participants and non-completers to completers. Inactive participants were significantly younger and tended to be significantly more likely to have a permanent partnership than active participants. In most of the studies carried out so far, partnership was not considered as a predictor of adherence, but marital status was examined more often (17). A meta-analysis showed that there was no association to adherence in 83% of the examinations. Two studies, on the other hand, found an association between an existing relationship and higher adherence (18, 36), with the main focus being on the additional social support from the partner, which was specified as a relevant factor. This is contrary to our findings. It is possible that participants in a permanent partnership felt less interested in the content of the program since they are in a close relationship and familiar with expressing feelings towards one another. Regarding age, the present result is that younger patients were less likely to start the intervention. This contradicts the findings of Christensen et al. (10) but is partially in line with previous studies (17), which found negative and positive correlations between age and adherence. One reason for these differing results could be that the structure of the intervention and the media used play a role in whether younger or older patients feel addressed. Some of the interventions that also found higher adherence among older participants were mainly text-based, similar to *KEN-Online* (37, 38). From a clinical perspective, it should be added that especially the younger of our patients often suffer from procrastination and deficits in coping with life (e.g., with precarious work and education situation), which could make it difficult for them to carry out a self-help program on their own responsibility on a regular basis. However, this was not investigated in this study. Furthermore, a significant difference in the diagnoses between the two groups could be determined, with significantly more comorbid diagnoses among inactive participants than among active participants. Inactive patients not only had significantly more comorbid disorders but also differed in terms of their diagnosis. In most of the studies published to date, diagnoses were not investigated because the studies did not take place in an inpatient setting or were aimed at a specific disorder, and the interventions were therefore not designed to be transdiagnostic. Regarding the severity of symptoms at the beginning of the intervention, it was also shown in our evaluation that inactive participants tended to have higher scores in symptoms of anxiety than active participants. Since there were generally more patients with a diagnosed anxiety disorder among the inactive patients in our study, higher anxiety scores among them are not surprising. Hence, especially at the beginning of the intervention, certain patient groups might not have felt addressed or might have felt overwhelmed by the intervention. Regarding the number of psychiatric diagnoses and symptom severity, there is no clear evidence yet on the impact on adherence (17). In the meta-analysis by Karyotaki et al. (16), it was reported that unaccompanied interventions led to a significantly higher drop-out risk for patients with depression, and Christensen et al. (10) found in a systematic review of internet-based interventions for anxiety disorders and depression that lower anxiety scores at the beginning of the intervention predicted higher adherence. Since the present intervention was not developed specifically for a certain disorder but is designed to be transdiagnostic, patients with comorbid anxiety disorder should also be able to identify with the content. Results that contradict with the findings of the present manuscript were observed in a study on a transdiagnostic internet-based relapse prevention for patients after inpatient treatment. Here, patients with anxiety disorder benefited significantly more from the intervention than patients with an affective disorder (39). Further research is necessary to understand the association between anxiety and high dropout rates in order to improve adherence to and efficacy of online interventions for this specific population.

The comparison between non-completers and completers showed no statistically significant differences concerning sociodemographic or symptom measures. However, the completers tended to have a higher score in terms of emotional competence.

Analyses of effectiveness showed a significant reduction of anxiety and depression in active participants in the course of their participation in *KEN-Online*, with particularly higher improvements in participants that completed more than half of the units. This result is similar to our prior study and to other studies that found associations between a higher adherence and higher efficacy of digital interventions (5, 11, 15). In addition, completers report higher emotional competence after participating in *KEN-Online* compared to the baseline assessment. This is gratifying, as this outcome best reflects the main objectives of *KEN-Online*.

### 4.1. Limitations

Since this was a naturalistic study, there are several methodological and statistical limitations. We had no control group and therefore our results concerning effectiveness must be viewed with caution, because they can also be a (long-term) effect of our inpatient/day-clinic treatment or other interventions (e.g., outpatient treatment) patients received afterward. Although all patients in the clinic should have been offered to use KEN-Online during inpatient or day-clinic treatment, we unfortunately have no systematic documentation, if this really is done and if patients really use it. Therefore we cannot make any statement about whether our sample is representative of the population of inpatient psychosomatic patients. Another constraint was that weekly assessments with the PHQ-4 were not mandatory; therefore, missing data was common over the course of the intervention. As we used the last available score of the PHQ-4 for our analyses, the statements on overall effectiveness and effectiveness over time use different time points for every participant in our sample. In some outcome measures concerning effectiveness (e.g., depression assessed with the PHQ-9) significance levels were only slightly exceeded and the effect size indicated that an effect could have been seen with a larger sample size. We also observed that we had many dropouts right at the beginning. We assume that the reason for this is that many patients were initially interested, but then did not take advantage of the program without further information about the use of *KEN-Online* or an integration of the intervention into regular treatment. A better integration of the digital intervention into regular treatment could be helpful here (39). This could be improved in future studies through training and greater involvement of therapists. In addition, another significant limitation was that the technical equipment and accessibility of *KEN-Online* were not optimal during inpatient or day-clinic treatment. Patients had to use private devices and patients without electronic devices could use written copies of *KEN-Online* during their clinic stay, which made inclusion in this study and weekly assessments impossible. There is a need for improvement here (e.g., wireless internet for patients and electronic devices like tablets), which could presumably increase adherence.

## 5. Conclusion

A strength of our study is its large, naturalistic sample of psychosomatic inpatients and day-clinic patients. Our main findings were that adherence outside of a clinical trial is lower than expected and there are only few patient characteristics (e.g., age, number of diagnoses, symptoms of anxiety) that differed between inactive and active participants. However, we could show that adherence is associated with greater effectiveness of our psychodynamic online self-help program. The aim of future research should therefore be to develop measures that can improve adherence, e.g., by promoting acceptance with acceptance-facilitating interventions or improving integration of digital interventions into regular treatment.

## Data availability statement

The original contributions presented in this study are included in the article, further inquiries can be directed to the corresponding author.

## Ethics statement

Ethical review and approval was not required for the study on human participants in accordance with the local legislation and institutional requirements. Written informed consent for participation was not required for this study in accordance with the national legislation and the institutional requirements.

## Author contributions

JB, AK, TS, and RZ developed the idea, concept, and design for the study. JB, AK, and RZ drafted the study manuscript. TS, AM, AT, and MB reviewed and revised the manuscript. All authors read and approved the final manuscript.
